# Prediction of bone ingrowth into the porous swelling bone anchors using an osteoconnectivity-based adaptive finite element algorithm

**DOI:** 10.1007/s11517-025-03370-6

**Published:** 2025-05-07

**Authors:** Amirreza Sadighi, Nolan Black, Mehrangiz Taheri, Moein Taghvaei, Sorin Siegler, Thomas P. Schaer, Ahmad R. Najafi

**Affiliations:** 1 Department of Mechanical Engineering and Mechanics, Drexel University, Philadelphia, PA 19104 USA; 2 Department of Clinical Studies New Bolton Center, University of Pennsylvania School of Veterinary Medicine, Philadelphia, PA 19348 USA

**Keywords:** Bone remodeling, Bone ingrowth, Co-polymeric bone anchors, Hygroscopic swelling, Osteointegration

## Abstract

**Abstract:**

In this study, a bone ingrowth framework was developed, which was integrated with a hygro-elastic swelling simulation, to evaluate the ingrowth of bone into porous co-polymeric swelling bone anchors. The aim was to investigate the impact of swelling-induced radial stress on bone ingrowth and the improvement in the mechanical properties and fixation strength of the anchors. Using the finite element method coupled with the osteoconnectivity matrix, the model successfully predicted the sequential bone formation within the porous bone anchor. The bone ingrowth framework was validated based on available experimental data, closely aligning with empirical observations. The results show that owing to radial stresses generated in the bone-anchor interface by swelling, considerable bone ingrowth could be stimulated. Moreover, among the three finite element models incorporating porosity within the recommended pore size range (300-600 $\mu m$), smaller pore sizes seem to promote faster and more extensive bone ingrowth, while larger pores exhibit slower ingrowth rates. Regardless of the pore sizes, the mechanical integrity and fixation strength of the anchors significantly improved. These findings strengthen the hypotheses that swelling of such anchors can stimulate bone ingrowth, and highlight the importance of pore geometry, size and interconnectivity in optimizing bone ingrowth and improving their performance.

**Graphical abstract:**

A quarter-slice finite element model of porous swelling bone anchors (with average pore sizes of 300, 450, and 600 μm) was developed, integrating a strain-energy-density (SED)-based bone ingrowth framework. This study examines how swelling-induced radial stresses at the bone-implant interface influence bone ingrowth. Additionally, mechanical integrity and push-out analyses were conducted to assess the role of bone ingrowth in implant fixation, considering added bone mass and filled void volume ratio. 
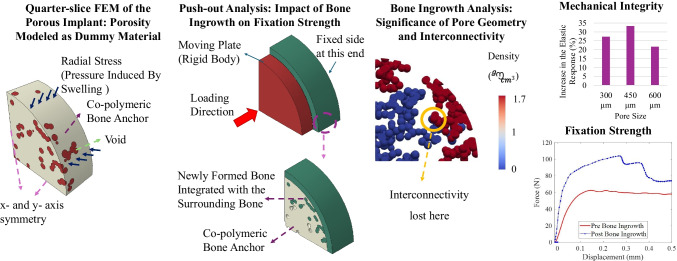

## Introduction

Suture anchors and screws are extensively utilized in orthopedic surgeries to ensure stable, long-term fixation. Common applications for these devices include reattachment of soft tissues to bone [1–5], as well as the repair of osteochondral defects and internal fracture fixation [6–9]. Despite their frequent use, bone anchors, particularly in low-density bones like osteoporotic bone, remain susceptible to failure due to the low resistance to shear forces [10–12]. Pullout failure of these anchors can lead to substantial damage in the surrounding bone, complicating the reattachment or repair process even further [13, 14].

Another reason for the failure of such anchors arises from the materials used, such as titanium, which has a much higher modulus of elasticity than bone. This mismatch in material properties can lead to stress shielding and, consequently, anchor loosening over time [15, 16]. In response to this issue, alternative materials with lower elastic moduli, closer to that of bone, such as PEEK (polyether ether ketone) and resorbable polymers, have been introduced [17–20]. Although these materials have advantages over traditional metals, threaded anchor designs made from them still face the same pull-out challenges as their metal counterparts [10, 11, 14].

To address these limitations, Greenberg et al. [21] were the first to investigate and develop a new type of swelling bone anchor. These innovative anchors absorb interstitial fluid and swell in situ, a process driven by a co-polymer composed of both hydrophobic and hydrophilic components [22, 23]. Unlike traditional anchors, which rely primarily on shear resistance, these swellable anchors achieve fixation through an expansion-fit mechanism [24]. As the anchor swells, it generates radial stresses on the surrounding bone, creating frictional resistance to pull-out forces [25–29]. Additionally, the locking mechanism observed in these anchors provides extra fixation [29].

The biocompatibility and osteointegration properties of these anchors have also been studied. Gualtieri et al. [30] conducted in-vivo and in-vitro research on these anchors and observed an increase in the bone density surrounding the anchor. This increase was attributed to the radial stresses acting as a mechanical stimulus, which promoted bone remodeling. Such remodeling is believed to be another factor contributing to the fixation strength of these anchors [31]. Unlike conventional threaded anchors, whose fixation strength predominantly depends on shear stresses developed between their threads and the surrounding bone, the fixation mechanism of porous swelling anchors relies primarily on frictional resistance at the interface. This frictional resistance increases gradually over time due to the radial expansion caused by the absorption of bodily fluids and subsequent bone ingrowth into the anchor’s pores. Consequently, these swelling anchors can effectively re-establish fixation to the surrounding bone even after exposure to significant dislodging forces [28]. Additionally, due to their friction-based fixation mechanism rather than thread engagement, swelling anchors cause minimal damage to the surrounding bone during pull-out, unlike traditional anchors which typically lead to substantial damage, as a cone-shaped portion of the bone fractures and is extracted along with the anchor.

Another crucial consideration in the design of bone anchors is the issue of stress shielding and its impact on bone remodeling [32]. Bone is a dynamic tissue that maintains mechanical and chemical equilibrium, adapting to the forces applied to it. Wolff’s law, first proposed by Wolff in 1892, suggested that bone structure is influenced by the mechanical forces it experiences [33, 34]. Bone remodeling involves the removal of bone mass in regions subjected to low mechanical loading, while new bone is formed in areas that experience higher mechanical stresses [35].

Although the precise relationship between mechanical loading and the activities of osteoblasts and osteoclasts is not fully understood, stress and strain-or more specifically, strain energy density (SED)-are commonly recognized as the mechanical signals driving bone remodeling [36]. Huiskes et al. [37] incorporated SED as a mechanical stimulus into their remodeling model, using it in conjunction with finite element analysis (FEA) to assess stress shielding and bone resorption in hip implants. Building on this, McNamara and Prendergast [38] investigated the impact of stress shielding in prostheses and applied a damage-based model developed by Prendergast et al. [39] to predict bone resorption around implants.

Bone resorption, which occurs when mechanical loading is insufficient, poses a significant challenge to the stability of implants during osteointegration [37]. Both underload (stress shielding) and overload have been implicated in bone resorption at the bone-implant interface. Huiskes and Nunamaker [40] observed that implant loosening and bone resorption were linked to high peak contact stresses during the early post-operative period. Van Oosterwyck et al. [41] developed a numerical remodeling algorithm to predict marginal bone loss caused by overload, defining an overload threshold for stresses. More recently, Crupi et al. [42] introduced a linear function to model the negative rate of bone density loss when stress exceeds a critical level, while Li et al. [43] and Liao et al. [44] incorporated quadratic methods into their remodeling models for improved accuracy in predicting bone density changes.

From a clinical perspective, long-term implant stability is influenced by several factors [45]. However, experimental methods for assessing implant survival and bone turnover rates are often limited by the radiation doses required for long-term image-based studies. Computational modeling, such as FEA, offers a non-invasive method to monitor bone remodeling over time, avoiding the challenges associated with animal studies and overcoming constraints such as time, cost, invasiveness, and ethical considerations [46, 47]. Additionally, measuring stresses around the bone-implant interface is challenging due to limited sensor space, which can interfere with the bone-implant contact area [48]. Finite element modeling is an effective, non-invasive method for predicting stress distribution in complex structures. It allows for the detailed assessment of strain and stress profiles throughout a structure and facilitates parametric analyses. Compared to experimental and clinical methods, FEM is a faster, more cost-effective approach for evaluating how implant design parameters affect the performance of the bone-implant system [49]. However, to better predict bone ingrowth progression into highly porous structures, standard strain energy density (SED)-based algorithms have shown limitations. To overcome these, a connectivity matrix was developed and incorporated in the remodeling cycle, allowing for the sequential formation of new bone around grooved implant surfaces, referred to as osteoconnectivity, which aligns more accurately with clinical timelines [50, 51]. This addition resulted in a close alignment between the clinical data of the bone ingrowth into the porous implants with the numerical results, signifying the importance of this modification.

To the best of the author’s knowledge, few studies have utilized bone remodeling algorithms to predict bone ingrowth into porous implants/bone anchors [50–52]. Specifically, in the case of swelling bone anchors and implants, it has been hypothesized that the swelling and the associated radial stresses induced at the bone-implant interface could stimulate both bone remodeling in the surrounding bone and bone ingrowth into the porous implant/bone anchor. However, no prior studies have demonstrated or reported this phenomenon. The aim of the current study was to develop an osteoconnectivity-based bone ingrowth framework to investigate new bone formation within porous swelling bone anchors.

In the current study, an already-developed and validated hygro-elastic framework was employed to simulate the swelling of bone anchors within the bone. Following the simulation, the average radial stress at the interface was extracted and applied as a loading condition in a finite element analysis (FEA) of bone ingrowth within the porous bone anchor. To capture this phenomenon, a bone ingrowth framework utilizing an osteoconnectivity matrix was developed to simulate the sequential formation of bone within the void regions of the porous anchor. To validate the accuracy of this framework, it was first applied to a finite element model (FEM) that replicated one already validated against clinical data from previous research [50, 51]. Upon achieving consistent numerical results, the framework was then applied to swelling bone anchor FEMs with varying pore sizes and geometries. Factors such as bone ingrowth rate, the impact of pore geometry and size, added bone mass, filled pore volume, enhanced mechanical integrity of the porous bone anchors, and the increased fixation strength due to bone ingrowth were studied and analyzed. The findings demonstrated that the radial stresses generated at the interface by the swelling promoted bone ingrowth and contributed to the osteointegration as well as the mechanical integrity of bone anchors. Additionally, the findings highlighted the importance of the interconnectivity of the pores for optimal bone ingrowth.

## Material and methods

### Swelling methodology

Hygroscopic swelling occurs when a solid is exposed to a moist environment, causing the material to absorb moisture and swell. When the structure is constrained, this swelling leads to the development of hygroscopic strains and stresses. These strains can be approximated assuming a linear relationship between moisture absorption and strain, given by the equation:

1 $$\begin{aligned} \varvec{\epsilon _{hs}} = \varvec{\beta _{h}} (\alpha _m - \alpha _{m,ref}), \end{aligned}$$

where $\varvec{\beta _{h}}$ represents the hygroscopic swelling coefficient tensor ($m^3/kg$), $\alpha _m$ is the current moisture content, and $\alpha _{m,ref}$ is the reference (initial) moisture content ($kg/m^3$) [53]. According to small deformation theory, the induced hygroscopic stress in the swelling bone anchor can be computed using the following linear elasticity equation:

2 $$\begin{aligned} \varvec{\sigma } = C\left( \frac{\nabla \varvec{u}^T + \nabla \varvec{u}}{2} - \varvec{\epsilon _{hs}} \right) , \end{aligned}$$

where $\varvec{\sigma }$ is the stress tensor, *C* is the elasticity tensor, and $\varvec{u}$ is the displacement vector.

The constrained swelling of the bone anchor generates compressive pressure at the interface between the bone anchor and surrounding bone (hereafter referred to as the bone-anchor interface), and induces tensile hoop stress in the surrounding bone. The compressive pressure at the interface contributes to frictional resistance against sliding, enabling the bone anchor to achieve fixation through an expansion-fit mechanism. Furthermore, the tensile hoop stress helps reduce stress shielding in the femur and stimulates bone densification near the bone anchor, potentially enhancing the remodeling process.

### Bone remodeling methodology

This study applied the traditional bone remodeling theory initially proposed by Weinans et al. [49] and Huiskes et al. [37], with modifications suggested by San Cheong et al. [50] and Cheong et al. [51], to be able to capture new bone formation. Mathematically, the remodeling process can be interpreted as an optimization problem, where the objective is to adjust the apparent bone density $\rho$ in response to the mechanical stimulus *S*. This is described by the following equation:

3 $$\begin{aligned} \frac{d\rho }{dt} = {\left\{ \begin{array}{ll} B(S - (1 + \delta )k), &  \text {if } S > (1 + \delta )k \\ 0, &  \text {if } (1 - \delta )k< S< (1 + \delta )k \\ B(S - (1 - \delta )k), &  \text {if } S < (1 - \delta )k \end{array}\right. } \end{aligned}$$

Here, *B* denotes the remodeling rate constant, *k* is the reference (homeostatic) stimulus under normal physiological conditions, and $\delta$ defines the “lazy zone”, which represents a threshold range where no bone remodeling occurs [32]. The first case indicates bone apposition/deposition, the second represents bone equilibrium, and the third signifies bone resorption. The mechanical stimulus *S* driving the process is defined as strain energy density, which is the ratio of strain energy *U* to the apparent bone density $\rho$ [54, 55]:

4 $$\begin{aligned} S = \frac{U}{\rho } \end{aligned}$$

The strain energy density *U* is defined for each element in the model based on the linear isotropic material hypothesis and small strain theory [54, 56], as follows:

5 $$\begin{aligned} U = \frac{1}{2} \varvec{\sigma }_{ij} \varvec{\epsilon }_{ij}, \end{aligned}$$

where $\varvec{\sigma }_{ij}$ and $\varvec{\epsilon }_{ij}$ are the stress and strain components at a material point, respectively.

As previously mentioned, the concept of “osteoconnectivity” is utilized to ensure sequential bone formation during each iteration. This is achieved through a large matrix that consists of sub-matrices for each element, where each sub-matrix records the neighboring element numbers. The osteoconnectivity matrix governs the bone remodeling algorithm, allowing only those elements adjacent to existing bone or previously remodeled elements to undergo densification, representing bone ingrowth, in subsequent iterations (as further explained in Section 2.4.2).

### Materials and design

This study models porosity of the co-polymeric bone anchors according to the manufacturing method in the previous research. The process has been extensively detailed in prior studies [23, 28, 29]. Accordingly, only a brief overview is presented here. The two monomers that underwent cross-linking were methyl methacrylate (MMA), which was described as hydrophobic and acrylic acid (AA) described as hydrophilic. Furthermore, to introduce porosity into the specimens, NaCl crystals with a random distribution were incorporated into the co-polymer mixture before polymerization. These NaCl crystals were removed at the end of the polymerization process through desalination in distilled water, leaving behind a porous swelling co-polymeric structure.

### Finite element models

#### Hygroscopic Swelling of Bone Anchors

Swelling simulations for the bone anchors were conducted using the commercial finite element software COMSOL Multiphysics 5.7, using the hygro-elastic framework explained in [29, 57]. The porosity in the finite element models was generated via a custom script imported into the software. This script randomly generated spheres with diameters ranging from 300 to 500 $\mu m$, a size considered optimal for promoting bone ingrowth and osteointegration based on previous studies [58, 59]. The bone anchors in the simulations had a fixed diameter and length of 8 *mm*, matching the dimensions of experimental samples used in earlier research (Fig. 2e) [28, 29].

To control the swelling rate, a co-polymer mixture with an 85/15 ratio of MMA/AA was employed in the finite element model (FEM). The material properties were derived from stress-strain curves of samples swelled in bovine serum, yielding an elastic modulus of 433 *MPa* in wet conditions and 479 *MPa* in dry conditions, with a Poisson’s ratio of 0.25 [23, 29]. Additionally, based on the weight and volume gain data from the free swelling experiments in bovine serum, the moisture concentration and swelling coefficient for the 85/15 swelling bone anchor were determined to be 0.08672 $kg/m^{3}$ and 0.9920 $m^{3}/kg$, respectively. Moreover, the density, elastic modulus, and Poisson’s ratio of the bone were set to be 0.8 $g/cm^3$, 1940.48 *MPa*, and 0.34, respectively [40, 49, 50, 57]. The details of this finite element analysis have been extensively discussed in previous works, and readers are encouraged to refer to [29] and [57] for further information.

At the conclusion of this simulation set, the average radial stresses throughout the swelling process were extracted to be used in the analysis of bone ingrowth. This average radial stress was determined using the “boundary probe” feature in COMSOL Multiphysics, a built-in function that allows for the post-processing extraction of surface load data from swelling simulations. The average radial stress ($\bar{\sigma }_{rr}$) at the bone-anchor interface was calculated according to the following relationship:

6 $$\begin{aligned} {\begin{matrix} {\bar{\sigma }_{rr} = \frac{\int _{A_{\text {interface}}} \sigma _{rr}\, dA}{A_{\text {interface}}}} \end{matrix}} \end{aligned}$$

where $\sigma _{rr}$ is the radial stress component normal to the interface, and $A_{\text {interface}}$ is the total bone-anchor interface area. This provides a representative average radial stress for subsequent finite element analyses.

**Fig. 1:**
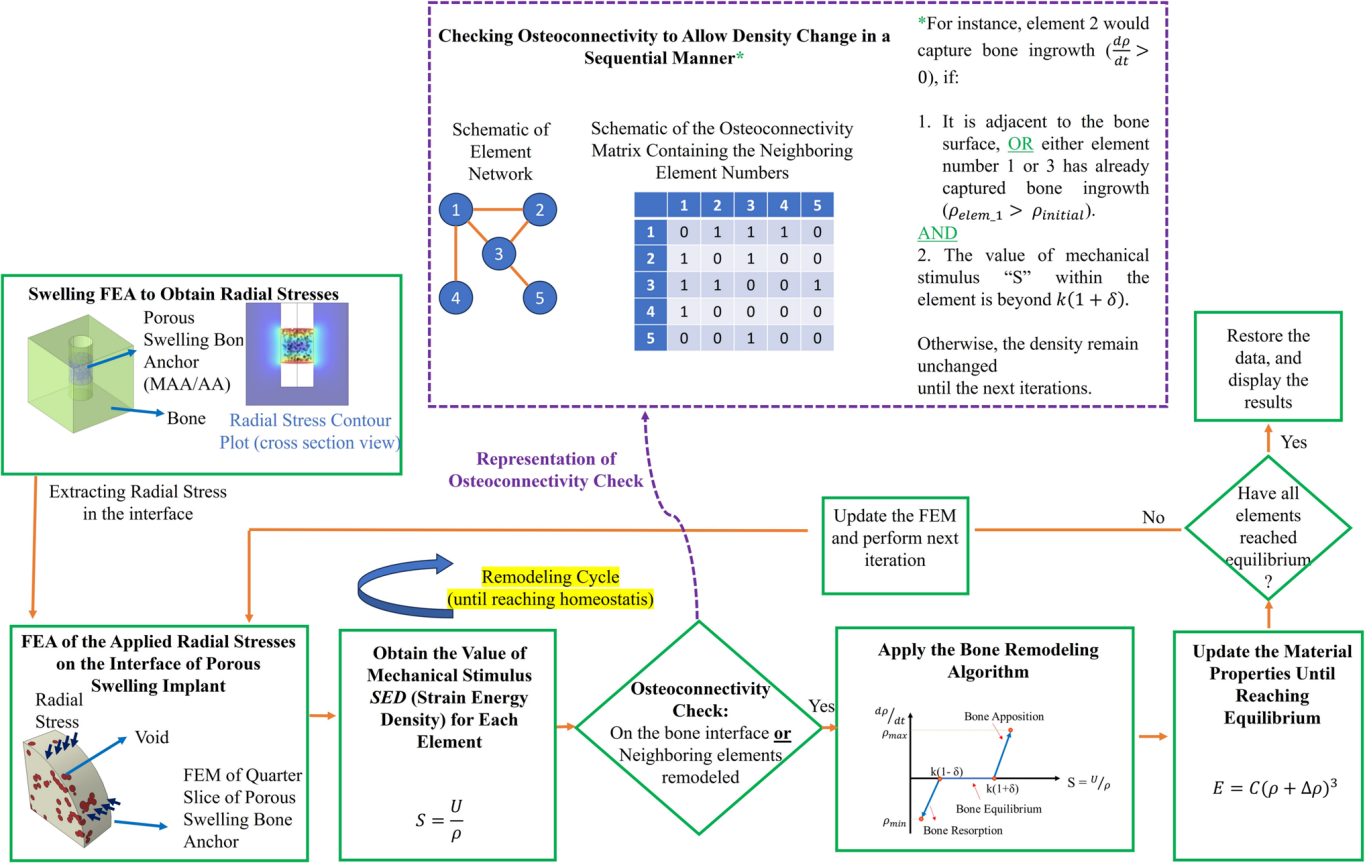
Illustration of the bone ingrowth framework process: After calculating the mechanical stimulus (S) for each element, the osteoconnectivity matrix was employed to determine whether bone ingrowth can occur in a specific element. This determination was based on the condition that neighboring elements experienced an increase in their densities, enabling sequential bone formation through the structure

#### Bone ingrowth framework

Assimilating finite element (FE) analysis and the remodeling algorithm provides a computational approach with which the underlying differential equation (Eq. 3) that governs the algorithm could be solved. In the FE analysis, elements can be considered numerous basic multicellular units (BMUs), containing mechanosensory cells that sense the strain energy density for each unit. Forward Euler time integration method was used to solve Eq. 3 as below,

7 $$\begin{aligned} \rho _{m}^{i} = {\left\{ \begin{array}{ll} \rho _{m}^{i-1} + B\Delta t (S_{m}^{i-1} -(1+\delta )k),&  \text {if } S_{m}^{i-1}>(1+\delta )k\\ \rho _{m}^{i-1}, &  \text {if } (1-\delta )k<S_{m}^{i-1}<(1+\delta )k\\ \rho _{m}^{i-1} + B\Delta t (S\rho _{m}^{i-1}-(1-\delta )k),&  \text {if } S_{m}^{i-1}<(1-\delta )k \end{array}\right. } \end{aligned}$$

where *i* represents the time step, and *m* is the bone element number. In the above-mentioned equations, the values for constant bone remodeling rate, *B*, was set to be 1.0 $(g/cm^3)^2(MPa\times time\,unit)$ $^{-1}$, as documented in previous literature based on the studies conducted on human bones [37, 49, 51]. Moreover, a value of 0.004 (*J*/*g*) was adopted for *k* as the reference for reference stimulus, aligned with the previous findings [37, 49], where it was reported above and below this level bone apposition and resorption occurs. The definition of the lazy zone around the reference stimulus involved setting $\delta$ at $10\%$ [35, 60, 61]. The algorithm iterated until there was no change greater than $2\%$ in the apparent density of each element, indicating bone homeostasis. Upper and lower bounds for density were set at 1700 and 10 $kg/m^3$, in the same order [37, 49, 61]. The parameter $\Delta t$, representing the time increment, was selected as 0.01 *s* based on previous research to ensure numerical stability and accuracy in bone remodeling simulations [61, 62]. Earlier studies utilized time increments ranging from $10^{-4}s$ to 1*s*, emphasizing that smaller increments enhance convergence robustness [37, 49, 61]. Our preliminary analyses confirmed that a conservative choice of 0.01 *s* improved stability and convergence to equilibrium (homeostasis), whereas larger increments led to excessive density changes and numerical instabilities (truncation errors).

The bone ingrowth analysis was conducted using Abaqus CAE through a Python script [63]. This analysis followed an iterative process, where each iteration could be interpreted as the passage of implantation time [45, 61, 64]. Figure 1 demonstrates the operation of the bone ingrowth framework. As previously explained, the average radial stresses at the bone interface were extracted at the end of the swelling simulations and imported into Abaqus as load input. The void region within the swelling bone anchor was modeled using a dummy material with relatively low stiffness. When the load was applied to the interface, the elements in the void section experienced stresses and strains, resulting in corresponding strain energy densities. However, unlike conventional bone remodeling algorithms, the density of elements did not increase automatically. Instead, the process was controlled by an osteoconnectivity matrix through the Python script. This matrix contained information on neighboring elements and ensured that only elements adjacent to the bone interface could capture bone ingrowth during the first iteration. Starting from the second iteration, the density of elements whose neighboring elements had captured bone ingrowth ($\rho _{i+1}>\rho _{initial}$) would also increase, simulating new bone formation/deposition in a sequential manner. A schematic of the element networks and the functionality of the osteoconnectivity matrix is provided in Fig. 1. According to this representation, element 3 is connected to elements 1, 2, and 5. Accordingly, based on the osteoconnectivity check, this element would be able to capture bone ingrowth if the density of elements 1, 2, and 5 increased in the previous iterations and provided that the mechanical stimulus of this element goes beyond the reference value ($k(1\pm \delta )$).

Additionally, this framework allows control over the rate of bone ingrowth. For instance, an element could be set to capture bone ingrowth only if the density of a neighboring element exceeded a specific threshold (*e.g.*, based on Fig. 1, if the density change track of element 1 indicated that “$\rho _{i}>1.25\times \rho _{initial}$”, implying a 25% increase at iteration $i^{th}$ from the initial configuration, then the adjacent elements (2, 3, and 4) could undergo densification). This would impact the bone ingrowth rate, postponing the occurrence of bone ingrowth in the adjacent elements until the density value of a particular element reached the specified threshold over several iterations.

**Fig. 2:**
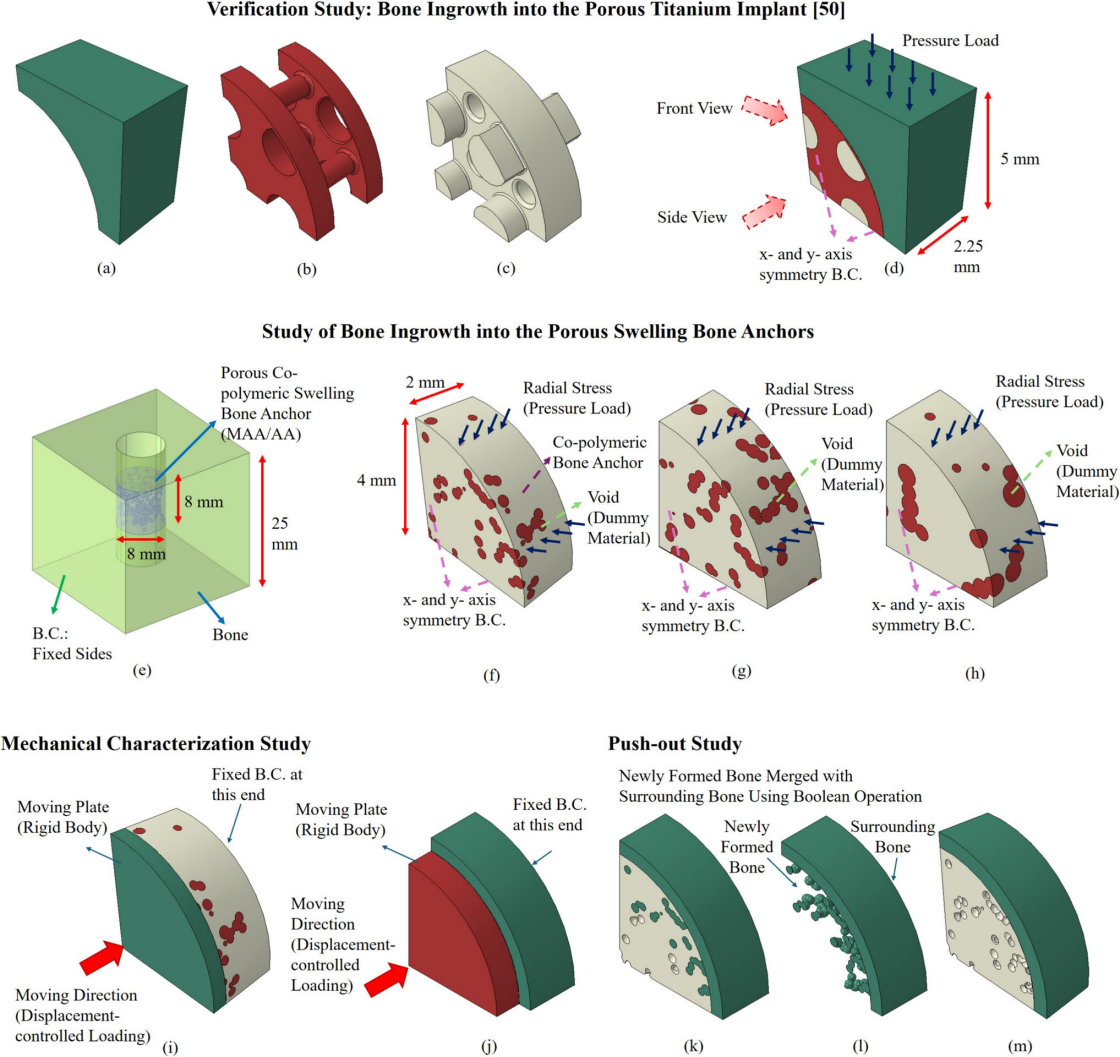
Representation of the FEMs; (a) Bone, (b) titanium implant, (c) void region between the bone and implant, (d) assembly of the FEM (replicating the FEM in [50]), (e) FEM of hygroscopic swelling (COMSOL), (f) FEM of porous swelling bone anchor with average pore size of 300 $\mu m$, (g) FEM of porous swelling bone anchor with average pore size of 450 $\mu m$, (h) FEM of porous swelling bone anchor with average pore size of 600 $\mu m$, (i) FEM of the mechanical integrity evaluation, (j) FEM of the push-out strength, (k) Post-ingrowth push-out FEM, (l) Post-ingrowth bone section (union Boolean operation of filled pores with the surrounding bone), and (m) Pre-ingrowth FEM. As quarter-slice FEMs have been used, symmetry boundary condition along x- and y-axes have been utilized

#### Bone ingrowth simulations within the porous region of titanium implant (verification of the framework)

To ensure the accuracy of the developed bone ingrowth script in this study, it was first applied to the design previously analyzed by Cheong et al. [50], where the bone ingrowth was experimentally and numerically evaluated within the porous titanium implants under physiological loading conditions (Fig. 2a-d).

This FEM consisted of a quarter-slice model of a porous titanium implant and the surrounding bone for computational efficiency, with a size of $5mm \times 5mm \times 2.25mm$. The top surface of bone was loaded by a uniform diametric pressure load of 200 N, corresponding to the peak axial force of 2.25 body weight (BW) in ovine stifle, and the x- and y-axes symmetry boundary condition was applied to the sides. The readers are referred to this study for further details regarding the FEM parameters and description. After validating the script, it was subsequently employed to simulate bone ingrowth into the void region of the porous swelling bone anchor. For computational efficiency, and similar to the FEM in [50], a quarter-slice of the model with a thickness of 2 *mm* was considered (Fig. 2f-h).

#### Bone ingrowth simulations within the porous swelling bone anchor

The void region within the porous swelling bone anchor was modeled using a dummy material with an initial elastic modulus of 0.05 *MPa* and a Poisson’s ratio of 0.3 [51]. This approach allows for finite element analysis (FEA) with minimal impact on the results, as the void region’s material properties are significantly lower than those of the swelling anchor. Bone ingrowth was represented by an increase in the density of each element.

Three different FEMs were developed for the porous swelling bone anchors, each with varying pore sizes: 300 ± 100 $\mu m$, 450 ± 100 $\mu m$, and 600 ± 100 $\mu m$. Throughout the manuscript, they are referred to as small, medium, and big pore size FEMs. These pore sizes were chosen to include the first two FEMs within the range of previously reported conducive sizes, while the third set represents larger pores that are generally considered too large for optimal bone ingrowth [58, 59]. Similar to the porous designs used in the swelling simulations in COMSOL, the pores in these FEMs were randomly generated using a custom script in Abaqus CAE. This approach was intended to mimic the experimental manufacturing process of the porous bone anchor specimens, which utilized NaCl crystals [23, 28]. However, this method does not ensure pore interconnectivity or provide a consistent interfacial surface (referred to as “pore throats” open to the surface [65, 66]) for the void section in the interface with bone.

Furthermore, the pressure load induced by swelling was extracted from the hygroscopic swelling simulations and imported into Abaqus FEA software. This load was applied using the "Pressure" load type with a time-dependent amplitude to accurately represent the distribution and evolution of pressure at the anchor interface throughout the simulation period. Additionally, symmetry boundary conditions were applied along the x- and y-axes of the finite element model.

Mesh convergence analysis required a fine mesh with a seed size of 0.05 *mm*, resulting in approximately 250,000 to 280,000 triangular quadratic elements, to ensure solution accuracy and properly capture the structural and material changes due to bone ingrowth. To maintain numerical stability and solution accuracy, the load was applied in small increments, starting at 0.001 and automatically reducing to a minimum increment size of $1 \times 10^{-10}$ when necessary.

#### Mechanical characterization simulations of the bone anchors

Following the bone ingrowth analyses, the mechanical integrity of all three FEMs of the porous swelling bone anchors was evaluated. For doing so, as shown in Fig. 2g, a displacement-controlled compressive load was applied to the anchors, and their elastic response (*i.e.*, the slope of the stress-strain curve) was assessed both before and after bone ingrowth. The objective of this FEA was to investigate the impact of bone ingrowth on the mechanical integrity of the swelling bone anchors. As before, symmetry boundary conditions were applied along the x- and y-axes of the model.

**Table 1:** Parameters used in the finite element models.

Parameter	Value	Unit	Reference/Citation
Pore sizes	300, 450, 600	$\mu m$	[58, 59]
Swelling coefficient ($\beta _h$)	0.9920	$m^3/kg$	[23, 29, 57]
Moisture concentration ($\alpha _m$)	0.08672	$kg/m^3$	[23, 29, 57]
Coefficient of friction (interface)	0.4	−	[69–71]
Bone remodeling rate constant (*B*)	1.0	$(g/cm^3)^2(MPa \times time~unit)^{-1}$	[37, 49, 51]
Reference stimulus (*k*)	0.004	*J*/*g*	[37, 49, 51]
Lazy zone width ($\delta$)	10%	−	[35, 60]
Density upper bound	1.7	$g/cm^3$	[37, 49, 61]
Density lower bound	0.01	$g/cm^3$	[37, 49, 61]
Time increment ($\Delta t$)	0.01	*s*	[61]
Elastic modulus (bone anchor)	479 (dry), 433 (wet)	MPa	[23, 29, 72]
Poisson’s ratio (bone anchor)	0.25	−	[23, 29, 72]
Elastic modulus (dummy material)	0.05	MPa	[50]
Poisson’s ratio (dummy material)	0.3	−	[50]
Elastic modulus (bone)	1940.48	MPa	[50]
Poisson’s ratio (dummy material)	0.34	−	[50]
Initial yield strength (*A*)	0.05	GPa	[67, 68]
Hardening modulus (*B*)	0.1	GPa	[67, 68]
Hardening exponent (*n*)	0.08	−	[67, 68]

#### Push-out simulations

Finally, the primary goal of using these swelling bone anchors-providing sufficient fixation to the surrounding bone-was examined. To do so, a layer of bone around the swelling bone anchor was included in the FEM (Fig. 2j-m), with the same symmetry boundary conditions applied along the x- and y-axes. A displacement-controlled push-out force with a magnitude of 0.5 *mm* was used to capture the peak push-out forces. For the FEA on the fixation strength without the bone ingrowth, only the boundary load in the interface provided by swelling would contribute to the fixation by creating frictional forces counteracting the push-out force, as depicted in Fig. 2m. However, in the case of bone ingrowth, the part of the void section that underwent densification, was merged to the surrounding bone through Boolean operation (Fig. 2k-l). Therefore, in order to push out the swelling bone anchor, apart from the frictional force, there was another contributing factor to the fixation strength, which was the force required to break the newly-formed bone in the void section from the surrounding bone. To model the damage in bone, aligned with the previous research [67, 68], a Johnson-Cook plasticity damage model was considered, where initial yield strength (*A*), hardening modulus (*B*), and hardening exponent (*n*), were set to be 0.05 (*GPa*), 0.1 (*GPa*), and 0.08 ,respectively. Moreover, erode feature was used to delete finite elements from the model when they experienced excessive deformations, ensuring numerical stability and a realistic representation of complete material failure (and their inability to bear loads).

8 $$\begin{aligned} \sigma _y = A + B \varepsilon ^n \end{aligned}$$

To enhance readability and reproducibility, the key parameters utilized in the finite element models and numerical simulations in this study are summarized in Table 1. This table provides a comprehensive overview, including parameter descriptions, numerical values, units, and corresponding references to ensure clarity regarding the model setups and facilitate future replication or verification of the analyses.

## Results

### Verification of the bone ingrowth framework

As discussed in Section 2.4, the bone ingrowth framework was validated using data from prior research [50]. This study was chosen because it validated a similar framework based on experimental findings. It is expected that our framework could predict the same bone ingrowth patterns observed in that study, and its validity would be demonstrated accordingly.

**Fig. 3:**
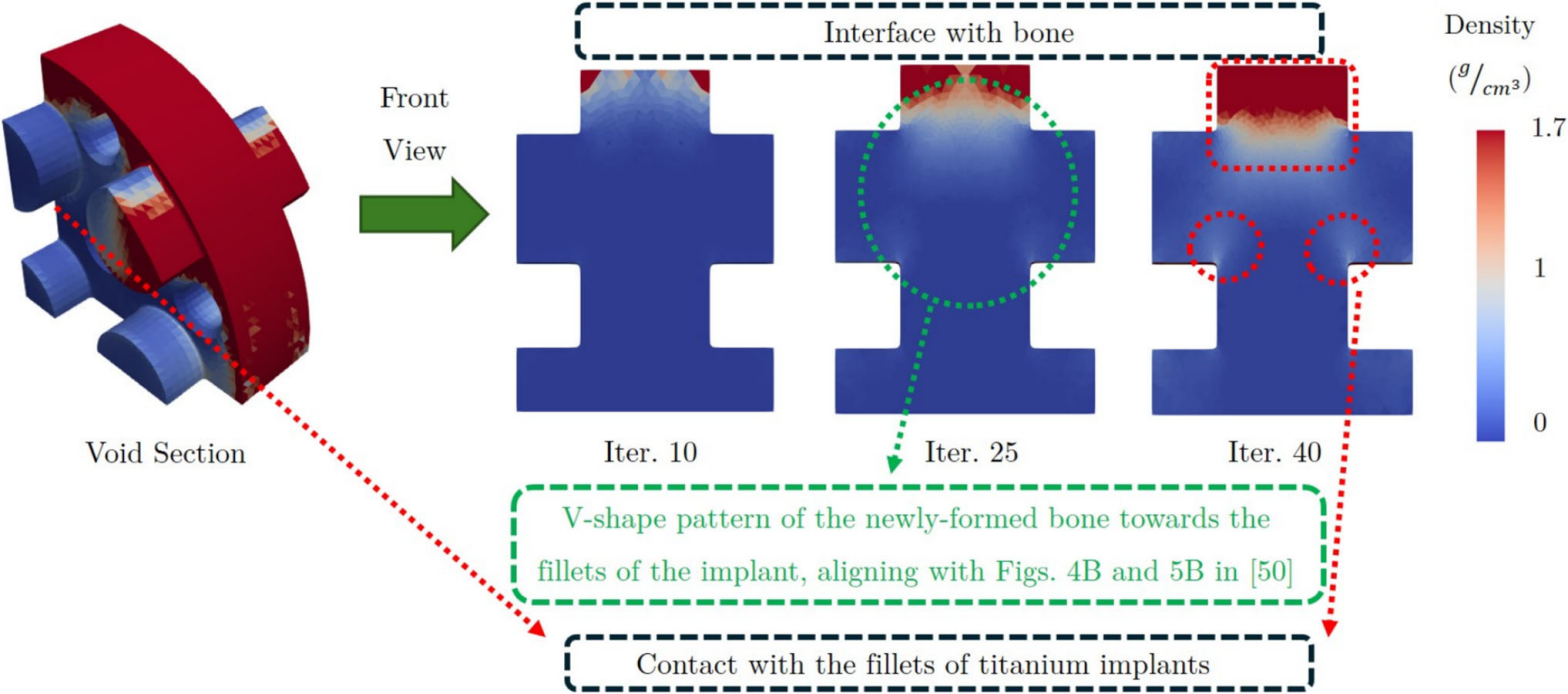
The bone formation in the void section. The new bone started forming from the interface with the bone, and then grew into the center of the void section. There was also bone deposition on the fillet of the bars of titanium implant as bone ingrowth progresses. The results were sensibly aligned with the validated results demonstrated in Figs. 4B and 5B of [50]

Figure 3 presents the newly-formed bone within the void section of the FEM (Fig. 2c). As observed, consistent with the reported results, the bone ingrowth initiated at the interface between the bone and the implant, gradually extending into the center of the void region. Additionally, in agreement with the findings reported by Cheong et al. [50], new bone deposition predominantly occurred around the fillets of the bars as ingrowth progressed (see Fig. 4B in [50]). Furthermore, consistent with the histological observations in the reference study (Fig. 5B in [50]), newly-formed bone preferentially expanded towards the fillets, resulting in a characteristic V-shaped pattern.

### Bone ingrowth into the porous swelling bone anchors

#### Bone ingrowth into the porous swelling bone anchors at different rates

Before investigating bone ingrowth, swelling simulations were performed to determine the average radial stresses generated at the bone-anchor interface, which served as input for the bone ingrowth analysis. The swelling of the bone anchor was found to induce average radial stress of 22 *MPa* at the interface [57]. This radial stress were then applied in the FEM for the bone ingrowth analysis.

The purpose of this section is illustrate the ability of the bone ingrowth framework to incorporate different formation rates. As explained in Section 2.4.2, with the developed osteoconnectivity-based framework, the rate of bone ingrowth could be controlled by determining a specific limit for the density of an element to reach before its neighboring elements in the void section could undergo densification ($\rho _{i}>\alpha \times \rho _{initial}$). To illustrate this, three cases of $\rho _{i}>\times \rho _{initial}$ , $\rho _{i}>1.25\times \rho _{initial}$, and $\rho _{i}>1.5\times \rho _{initial}$ as relaxed, moderate, and strict constraints, respectively. For instance, the first case would denote that bone can grow into cellular units (here modeled as elements) as soon as it grows into its neighboring cells, whereas the strict constraint would mean that not until the density of the bone grown within a cell increases by 50% would it grow into the neighboring cells.

**Fig. 4:**
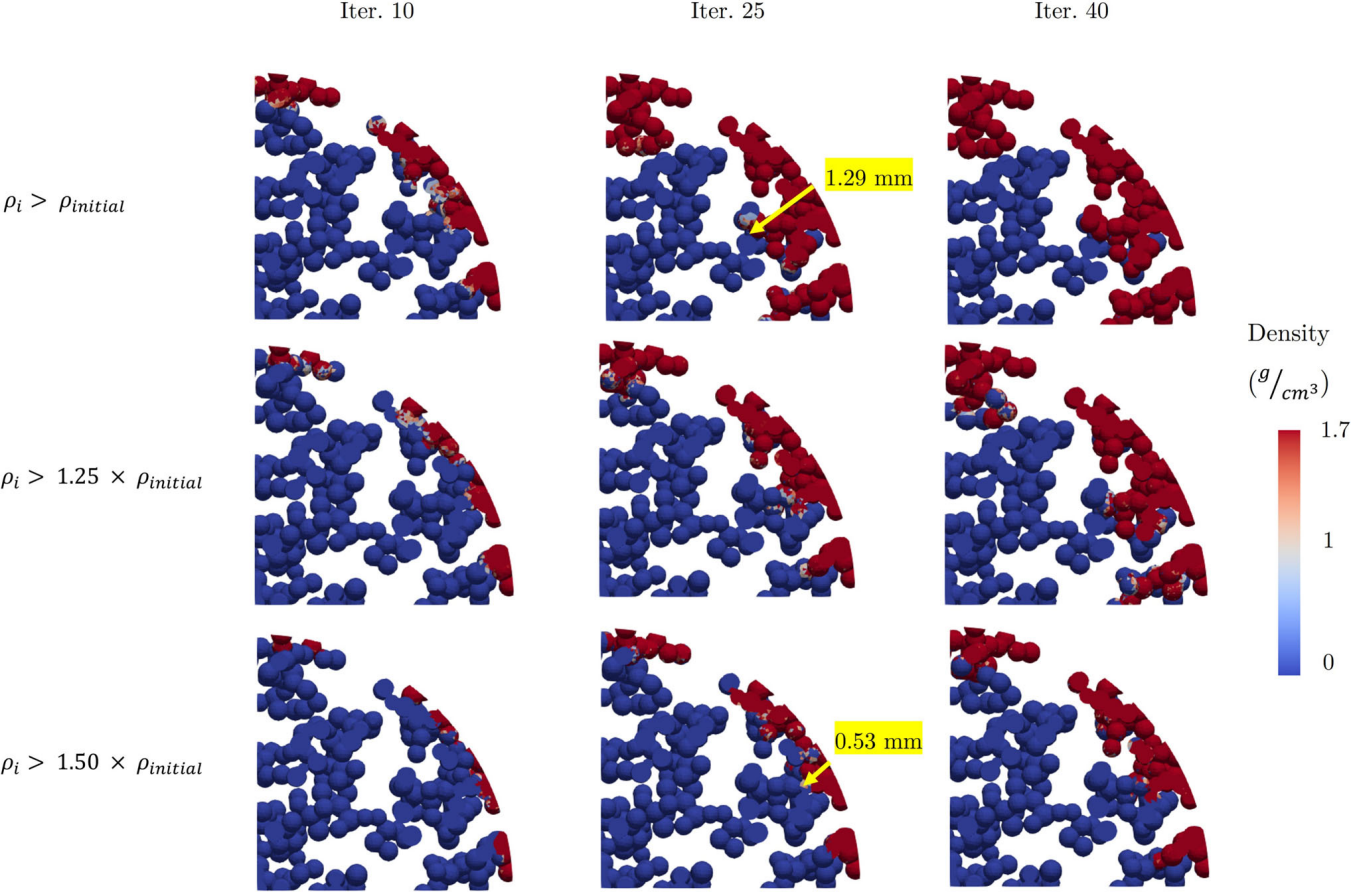
The side view of the void section with average pore size of 300 $\mu m$. The rate of bone ingrowth is constrained considerably when the limit of $\rho _{i}>\alpha \times \rho _{initial}$ is more strict

Figure 4 shows the progress of bone growth into the void section within the porous swelling bone anchor with the average pore size of 300 $\mu m$ for different growth rates. As it can be seen, when the limit was set to $\rho _{i}>1.5 \times \rho _{initial}$, where $\rho _{i}$ was the density of a neighboring element in the current iteration, the ingrowth rate was considerably different from the case of $\rho _{i}>\rho _{initial}$, where the constraint was relaxed. As an illustration, at iteration 25, the depth of bone ingrowth for the relaxed constraint of $\rho _{i}>\rho _{initial}$ was 1.29 *mm*, whereas this value for the strict constraint of $\rho _{i}>1.5 \times \rho _{initial}$ was 0.53 *mm*, representing a decrease of nearly 69%.

#### Bone ingrowth into the porous swelling bone anchors with different pore sizes

As explained in Section 2.4.2, in this section, the progression of bone ingrowth was examined in the void regions of bone anchors with three distinct average pore sizes of 300, 450, and 600 $\mu m$. The objective was to assess whether variations in pore size would influence the extent or rate of bone ingrowth. For all pore sizes, the osteoconnectivity criterion of $\rho _{i}>\rho _{initial}$ was set to allow for more flexible growth patterns.

**Fig. 5:**
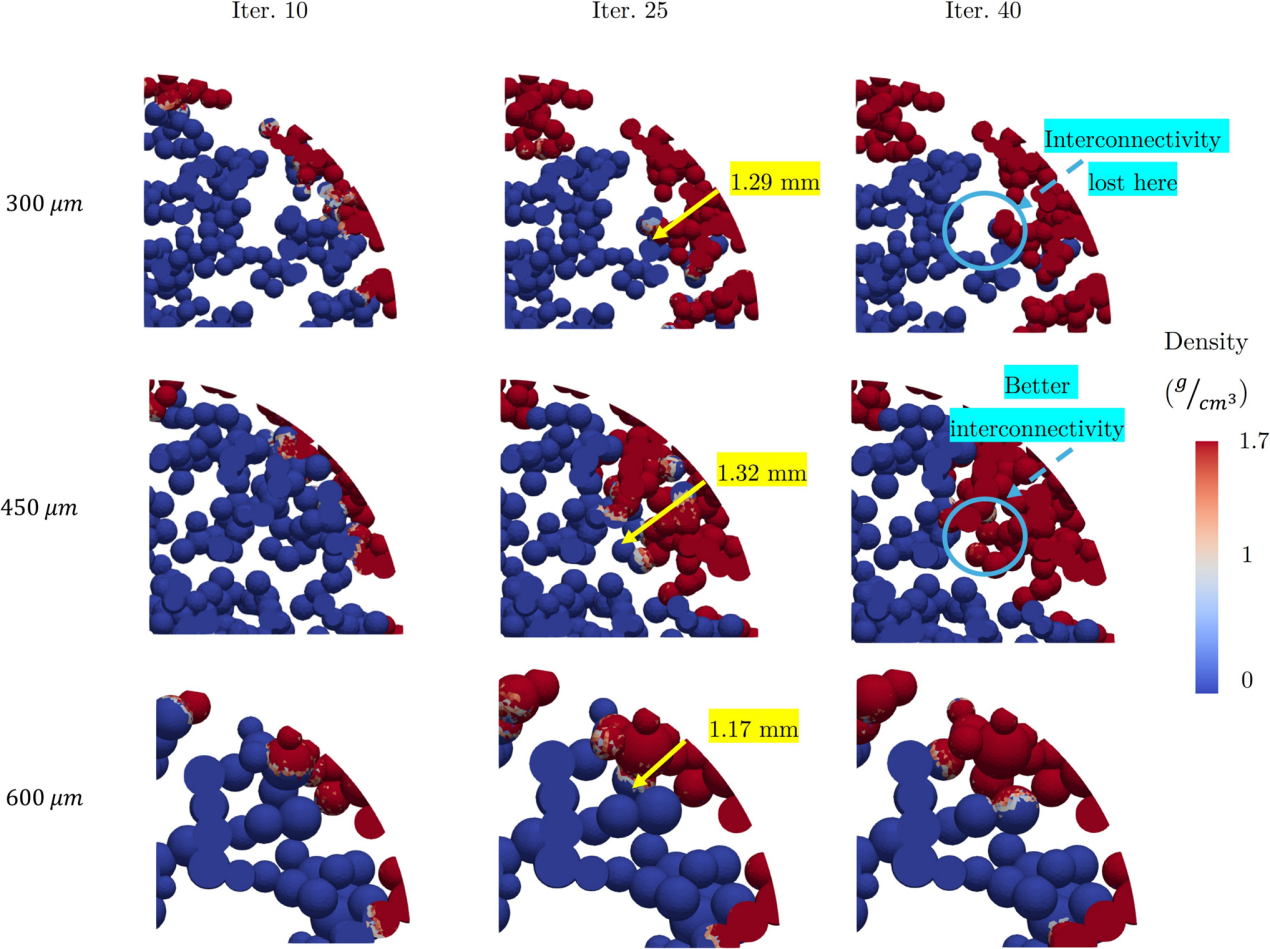
The side view of the void section with different average pore sizes of 300, 450, and 600 $\mu m$. The rate of bone ingrowth seemed to be slower for the big pore size. The interconnectivity of the pores played an important role in the progression of the bone ingrowth

Figure 5 presents the formation of new bone within the void regions for the specified pore sizes. As observed, significant bone ingrowth was initiated by the swelling of the bone anchor and the resultant radial stresses at the bone-anchor interface. Nevertheless, the rate of bone ingrowth appeared slightly reduced for the big pore FEM compared to the medium and small pore ones. Furthermore, when comparing the small and medium pore sizes, it was evident that pore interconnectivity plays a crucial role in promoting bone formation. To illustrate, while bone ingrowth rates for the small and medium pore sizes seemed to be similar by the $25^{th}$ iteration, the superior interconnectivity of the FEM with medium pore size resulted in deeper bone penetration toward the center of the void in subsequent iterations.

**Fig. 6:**
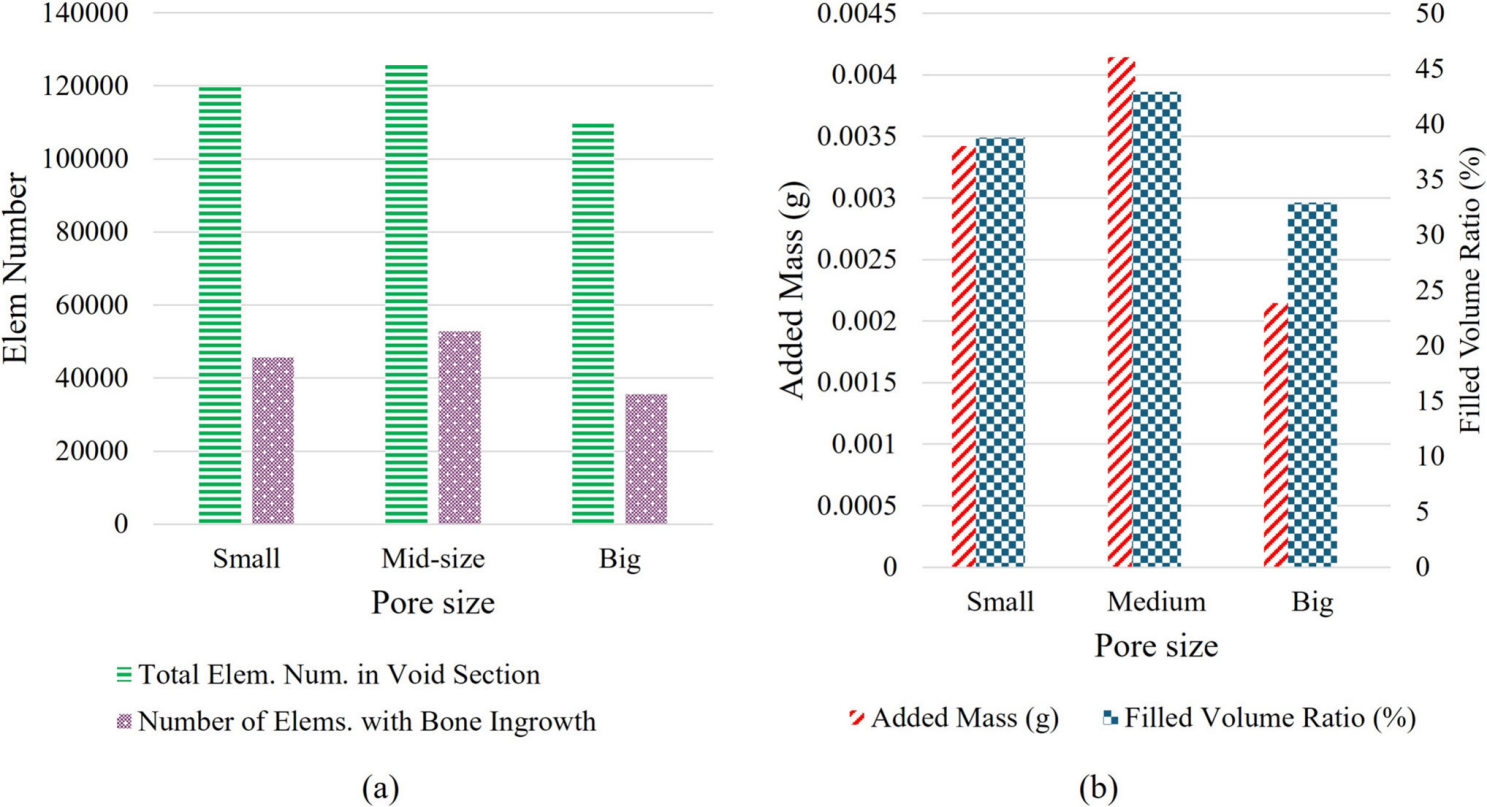
(a) The number of elements with the bone ingrowth, and (b) the added mass and filled volume ratio for each model. Considerable volume of void section for all three FEMs were filled with the new bone. Interconnectivity and pore size seem to have an impact on the amount of filled volume ratio and added mass

To better understand the extent of bone ingrowth, Fig. 6 is provided. As shown, with a porosity of approximately 60% for all FEMs, the number of elements in the void region ranged from 115k to 125k. The number of elements where density has increased, indicating bone ingrowth, was 56k, 58k, and 47k for the small, medium, and large pore size FEMs by the $40^{th}$ iteration, respectively. The filled volume ratios in the void region for these models were approximately 39%, 43%, and 33%, while the corresponding added masses were 0.0035, 0.004, and 0.0021 *g*, in the same order.

**Fig. 7:**
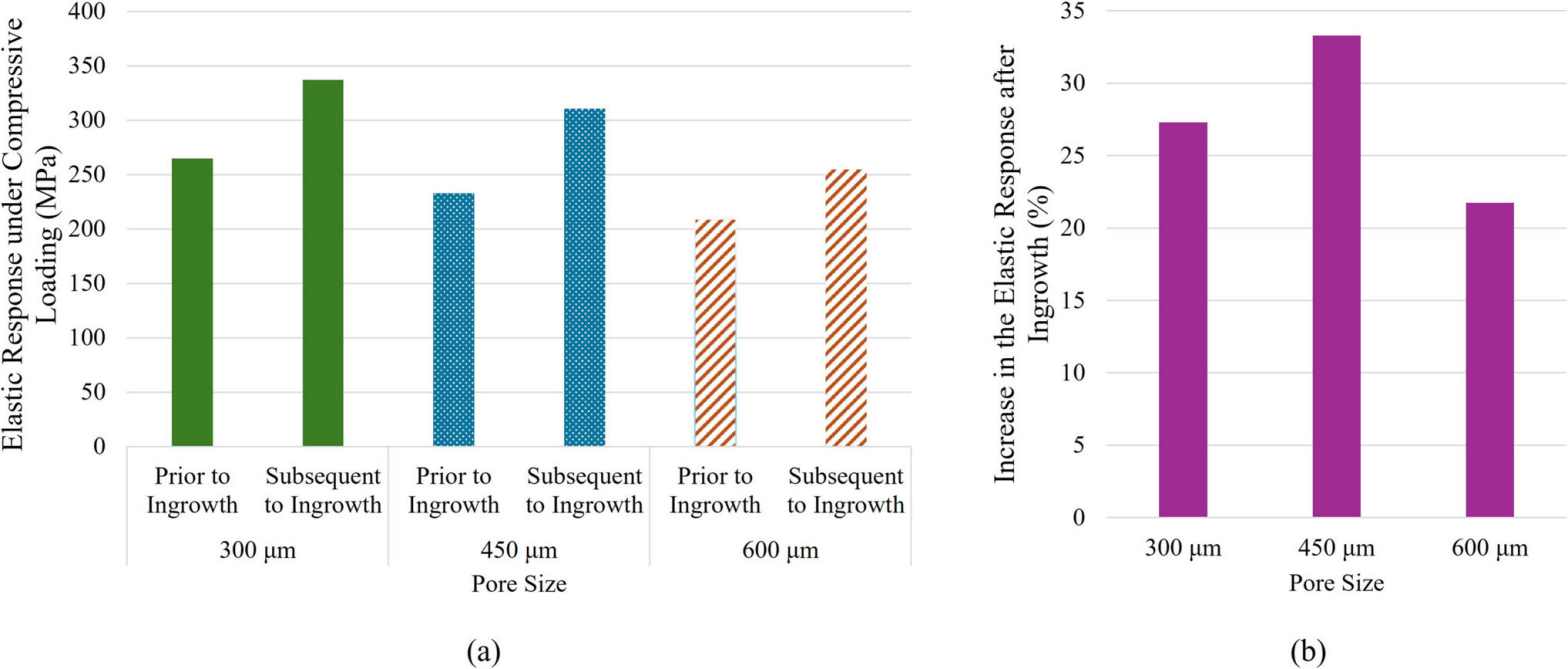
(a) The FEM of the mechanical characterization analysis, (a) The elastic response of the swelling bone anchors with different pore sizes prior and subsequent to the bone ingrowth, and (b) the increase in the elastic response due to the bone ingrowth for different pore sizes. The bone ingrowth induced by the swelling can considerably increased the elastic response

**Fig. 8:**
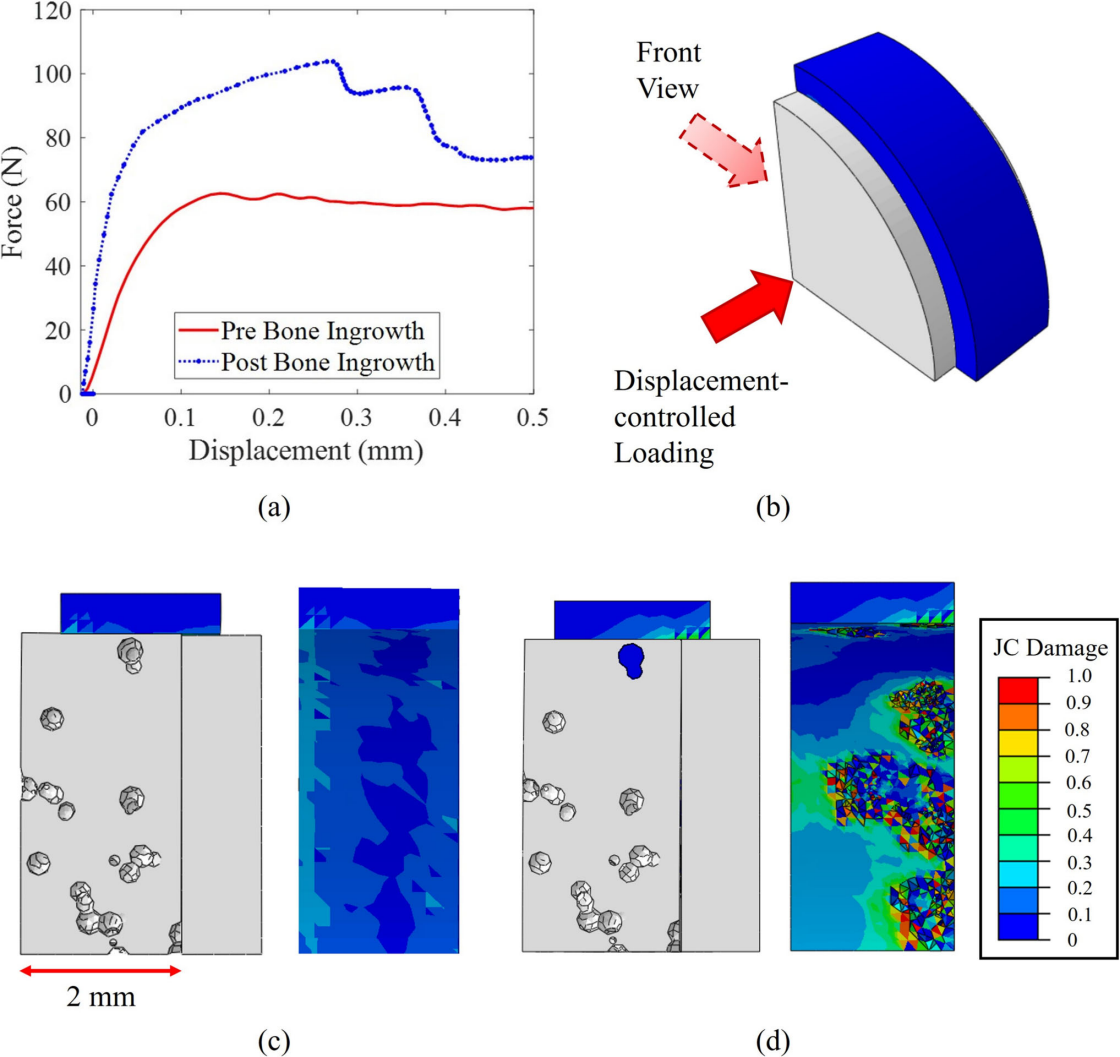
(a) The FEM of the push-out of the swelling bone anchor, (b) the FEM with the bone ingrowth, (c) the surrounding bone as well as new bone formation merged as one part, (d) the FEM with prior to the bone ingrowth, (a) The force-displacement result of the push-out FEA, (b) the isometric view of the FEA of push-out, (c) the JC damage controur plot of the FEA of push-out prior to the bone ingrowth (front view), and (d) the JC damage controur plot of the FEA of push-out subsequent to the bone ingrowth (front view). Bone ingrowth significantly affected the fixation strength, as the newly-formed bone had to break away from the bone-anchor interface

#### Impact of bone ingrowth on the mechanical integrity of swelling bone anchors

In this section, the material properties of porous swelling bone anchors with varying pore sizes were investigated before and after the onset of bone ingrowth to assess its impact on the overall mechanical response of the anchors.

For a fair comparison, the mechanical integrity analysis was applied to the FEMs with bone ingrowth properties (densities) within the void section at the $40^{th}$ iteration. The reason is that at this point the pores were interconnected in all FEMs. However, beyond this point, because of the non-uniform interconnectivity among the FEMs, some models would capture more ingrowth (deeper towards the center), while others would stay unchanged.

A key observation when pre-ingrowth results are considered is that pore size alone has a minimal effect on the mechanical integrity of the swelling bone anchors integrity of the swelling bone anchors (Fig. 7). Prior to bone ingrowth, the small pore size model exhibited a slightly higher elastic response, though the difference was not substantial. This is reasonable, given that the porosity across the three FEMs was comparable. However, the progression of bone ingrowth resulted in a notable increase in mechanical integrity across all models, with improvements of 27%, 33%, and 22% for the small, medium, and large pore size FEMs, respectively. Additionally, the disparity in elastic response between the models became more pronounced after bone ingrowth. The small and medium pore size models showed an increase in elastic response that was approximately 5% and 11%, respectively, higher than that of the large pore size model, underscoring the significant role of pore size in enhancing the mechanical performance of the structure following bone ingrowth.

#### Impact of bone ingrowth on the fixation strength of the bone anchors

In this section, the impact of the bone ingrowth was investigated on the ultimate goal of using swelling bone anchor, which is providing fixation to the surrounding bone. As explained in Section 2.4.2, two different FEMs, one for the pre-ingrowth analysis and the other for post-ingrowth, were studied to understand the impact of bone ingrowth on the added fixation.

As it can be seen in Fig. 8a, the bone ingrowth could cause a considerable difference in the magnitude of fixation strength. Based on the force-displacement result, the maximum push-out force for the case of pre bone ingrowth was around 62 *N*, which is the force to overcome the frictional resistance in the interface. However, this value for the case of post bone ingrowth was approximately 103 *N*, which included the frictional term as well as the force required to break the new bone formation from the interface. Moreover, it is noteworthy that this value corresponds with the current FEM which only considered a 2 *mm* thick bone anchor (Fig. 8c). By the use of a thicker bone anchor, similar to the ones in experimental investigations which were around 8 *mm*, it is expected that the difference between the fixation strength of cases pre- and post-ingrowth would become even greater. The reason is that in the latter case, there would be more bone-anchor interface and the associated frictional resistance, as well as more pore throats and ingrowth, which would require a higher force to break.

## Discussion

The current study developed and validated a bone ingrowth framework tailored for porous swelling bone anchors, which expands upon existing bone remodeling algorithms by incorporating the novel osteoconnectivity matrix. This matrix was integral in accurately predicting the bone ingrowth sequence, which allowed bone formation to follow clinical patterns of gradual progression from the bone-anchor interface into the center of void region. The framework demonstrated validity when compared to prior experimental findings, specifically those of [50], indicating its robustness in simulating realistic bone remodeling phenomena.

**Fig. 9:**
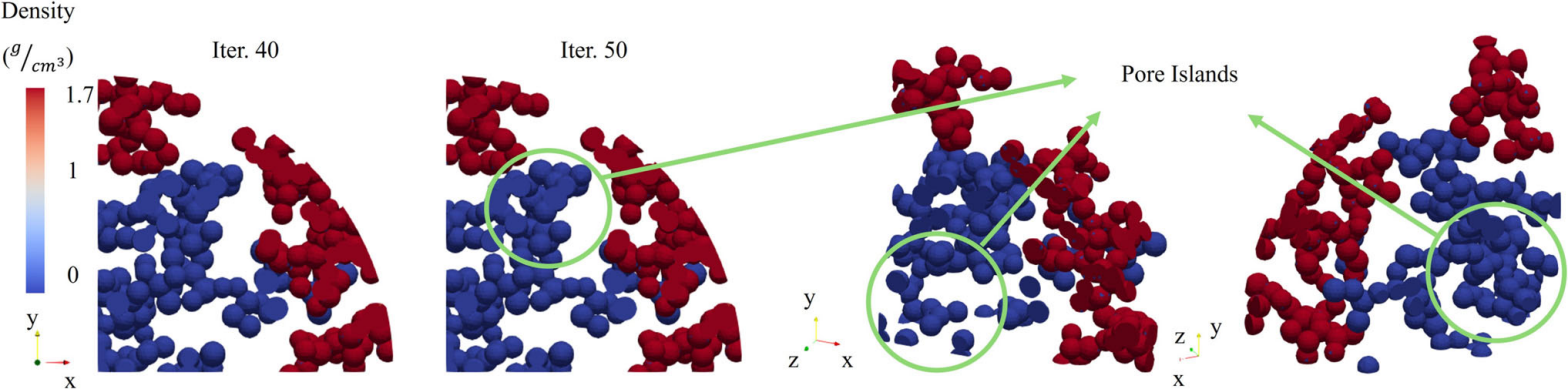
A representation of how pore islands (pores without interconnectivity) would both inhibit the progression of bone ingrowth, while also degrading the mechanical integrity of the swelling bone anchors. No more pores captured bone ingrowth beyond iteration 40 as the pores were not interconnected

The investigation into the bone ingrowth progression for various pore sizes (300, 450, and 600 $\mu m$) revealed that pore interconnectivity plays a critical role in promoting bone growth. The results suggest that smaller pore sizes lead to faster and more extensive ingrowth. Particularly, beyond the 25th iteration, because of the higher and better interconnectivity of the FEM with medium-sized pores, more significant central penetration of new bone tissue was observed. However, the larger pores exhibited a reduced ingrowth rate. This is likely due to having more number of elements in each pore, resulting in a bigger neighboring element sub-matrix for each element, which would further control the ingrowth rate into the adjacent pores. However, less interconnectivity and inconsistency in the number of pore throats to open surface could also be the factor that delayed the progression of new bone formation. As the pores were created by the random distribution of the spheres and no control would be possible through this method on the interconnectivity of the pores, therefore for a direct comparison and conclusion about the impact of pore size on the bone ingrowth rate and amount, further investigation must be conducted. Some additional FEA have been conducted in Appendix A, and the results and impact of pore size and interconnectivity were further discussed.

However, as explained above, apart from ensuring to encompass a sufficient porosity within the bone anchors and implants, its method of creating also plays a crucial rule. As we presented in this paper, the method of random distribution of NaCl crystals used in the experimental investigations [23, 28] would not allow for any control over the interconnectivity of the pores. Like the results in Section 3.2.2 showed, in the case where the pores were not interconnected, after a specific iteration, which represents implantation time in bone remodeling framework, bone ingrowth would be inhibited, and there would be “pore islands”. In this case, they were not able to capture bone ingrowth, and they only caused a drop in the mechanical integrity of the structure (Fig. 9). Moreover, it is important for the external surface of the porous bone anchor to have sufficient interface and pore throats with the surrounding bone to allow for bone ingrowth. However, this factor cannot be ensured through this manufacturing method either. These observations suggest that the method of creating porosity in the swelling bone anchors needs to be revised to ensure interconnectivity. These findings align with previous research highlighting the importance of pore geometry in bone ingrowth and osteointegration [58, 59], as well as the presence of sufficient pore throats [65, 66].

In Section 3.2.1, various constraints affecting the ingrowth rate were explored. This developed framework allows for control over the ingrowth rate by determining when (at what iteration) the neighboring elements can capture bone ingrowth. This control is achieved by applying the constraint “$\rho _{i}>\alpha \times \rho _{initial}$” in the Python script before the start of bone remodeling cycle in iteration $i^{th}$. In this constraint, $\alpha$ can be any number greater than 1, which increases the strictness of the constraint. To the best of the authors’ knowledge, there is no existing study that specifies the density a point in the bone matrix (osteoid) must reach before adjacent its neighbor can densify. Therefore, this parameter can be further adjusted and validated against clinical data over time in future investigations.

In Section 3.2.3, it was observed that the bone ingrowth would cause a significant recovery in the reduced mechanical integrity of the bone anchor, which would be caused by incorporating porosity [23]. As it was seen, regardless of the pore size, the bone ingrowth would cause a leastwise 20% increase in the load bearing capacity of the bone anchor in case it undergoes biomechanical daily loads. This recovery could even be greater in case the pores were interconnected and the bone ingrowth progressed further towards the center of the bone anchors. This observation is in alignment with the findings in [73], where it was seen that bone ingrowth would give rise to the mechanical strength of the anchors. It also confirms the results of the previous research where it was reported that the integration of bone into porous anchors enhances mechanical stability [58, 74].

In Section 3.2.4, the impact of the bone ingrowth on the increased fixation strength was studied. As it was seen, there can be a considerable difference between the fixation strength when an appropriate porous design allows for bone ingrowth. In the example which we investigated, there was a significant 62%increase in the fixation. It is indeed to acknowledge that due to the method of creation of porosity, this added fixation strength can be different from one design to another, depending on the interconnectivity of the pores and how many pore throats exist on the external surface of the bone anchors. This observation is in agreement with the previous research, where through push-out/pull-out tests, it was proven that there can be a significant rise in the fixation strength due to a porous design which allows bone ingrowth [75]. This considerable increase in the fixation strength, which could go beyond the reported magnitude knowing that we considered only a quarter slice FEM and 2-*mm* thickness to decrease computational cost, agrees well with the previous research, including the reports by [76] and [77], where the fixation strength of the porous bone anchors was tested by push-out tests and were reported to increase by 50 to over a 100% depending on the implantation period and porous system.

In the end, some limitations of this study must be acknowledged. The study incorporated a quarter slice FEMs and applied x-axis and y-axis symmetry to the sides, knowing that due to the random distribution of pores our problem was not symmetrical. However, this assumption was necessary as due to the nature of bone remodeling analysis. Such simulations are iterative, and with the very fine mesh network and high element numbers throughout the pores, which significantly dominate the duration of each bone remodeling cycle, the computational cost would be very high otherwise. Another limitation is that only three FEMs with the different pore sizes were considered. Therefore, a comprehensive investigation must follow this analysis, performing a parametric study on a higher number of FEMs with different pore sizes while keeping other factors (porosity ratio, pore interconnectivity, pore throats, etc.) the same to be able to conclude how the pore size would impact bone ingrowth from the perspective of this framework.

## Conclusion

This study incorporated a numerical framework for simulating bone ingrowth into porous co-polymeric swelling bone anchors using FEM and an osteoconnectivity-based bone remodeling algorithm. It was observed that owing to the radial stresses in the bone-anchor interface induced by the swelling, bone ingrowth is stimulated through the pores. The findings also demonstrated that bone ingrowth significantly enhances both the mechanical integrity and fixation strength of the anchors. Small and medium pore sizes seemed to have superior performance in terms of bone ingrowth and mechanical improvements. This validated model presents a valuable tool for future investigations into optimizing bone anchor designs, especially those involving swelling mechanisms.

## Figures and Tables

**Fig. 10:**
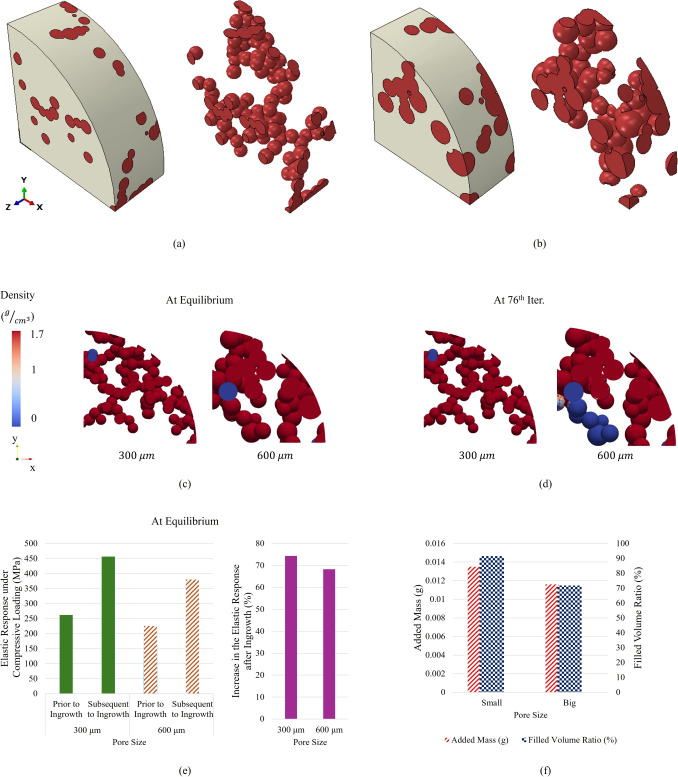
(a) The FEM with small pore sizes (300 ± 100 $\mu m$), (b) the FEM with big pore sizes (600 ± 100 $\mu m$), (c) the representation of bone ingrowth at equilibrium, (d) the presentation of bone ingrowth at iteration $76^{th}$ (ingrowth reaching equilibrium in the FEM with small pores while still in progress for the one with big pores), (e) the increase in the mechanical integrity of the swelling porous bone anchors after reaching equilibrium, and (d) the added mass and filled volume ratio for the FEMs

## Appendix A: Further investigation of impact of pore size on the bone ingrowth rate

Following the analysis of bone ingrowth into the porous swelling bone anchors, it was observed that the small and medium pore size FEMs captured more bone ingrowth compared to the large pore size FEM. In this appendix, two new FEMs were created-one with small pore sizes (300 ± 100 $\mu m$) and another with large pore sizes (600 ± 100 $\mu m$)-with two objectives: (i) to determine if the same trend of bone ingrowth is observed in these FEMs, and (ii) to ensure the interconnectivity of the pores in these FEMs and assess how this impacts mechanical integrity.

The two newly generated FEMs are shown in Fig. 10a-b. The pore networks in these FEMs are interconnected through the center of the void section, enabling continuous bone ingrowth. Figure 10c illustrates the progression of bone ingrowth until equilibrium is reached for all elements. As shown, the interconnectivity of the pores facilitated bone ingrowth towards the center of the void section. However, for a fair comparison, Fig. 10d presents the bone ingrowth progression at equilibrium for the FEM with small pore sizes, while growth is still progressing for the FEM with large pore sizes. This observation reinforces the results in Section 2.4.2, where small and medium pore size FEMs demonstrated higher bone ingrowth rates than those with large pores. Despite the interconnectivity of the pore networks in both FEMs here, still the pore throats and number of channels through the center are not the same in the two FEMs due to the completely random distribution of the pores. This introduces some uncertainty in drawing definitive conclusions. Therefore, as a future direction, a method for generating pores that ensures consistent conditions across different pore sizes should be explored to investigate the effect of pore size on ingrowth rates with greater confidence.

Figure 10e-f are also noteworthy. As shown, regardless of pore size, both FEMs exhibit a significant increase in elastic response post-ingrowth. Previously, the lack of pore interconnectivity limited the mechanical recovery of the porous bone anchors due to constrained bone ingrowth. However, when bone was able to grow all the way to the center of the porous anchor, there was a substantial increase in mechanical integrity. Additionally, the filled volume ratio and added mass were significantly higher than those reported in Section 3.2.2, thanks to the pore interconnectivity and bone ingrowth progression towards the center. This finding highlights the importance of pore geometry in bone anchors and the need to ensure their interconnectivity.
